# Rheumatic illness: transition from pediatric to adult care, patient satisfaction before and after the transition

**DOI:** 10.1186/1546-0096-12-S1-P105

**Published:** 2014-09-17

**Authors:** Annunziata Officioso, Claudia Traverso, Nicoletta Bertolini, Sara Cotini, Mariangela Raimondo, Nadia Scotti, Maria Alessio

**Affiliations:** 1 Department of pediatrics, Federico II University, Naples, Italy; 2 Department of pediatrics, Federico Ii University, Naples, Italy; 3 Department of Clinical and Experimental Medicine, Federico Ii University, Naples, Italy

## Introduction

There is increasing focus on the problems involved in the transition and transfer of young adult patients, from paediatric to adult rheumatic units, because transition is a critical process in the life of patients.

## Objectives

To assess patient satisfaction and quality of life from the “patients perspective”, before and after the transition from paediatric to adult care.

## Methods

Patients: Participants included 50 patients (M=12, F=38), matched for social class were asked to answer questionnaires for satisfaction end to fill PGWBI test.

This observational study had two phases. Prior to transfer pediatric patients to adult care, the subjects were asked to fill out a closed-question questionnaire addressing patients’ satisfaction and PGWBI to assess their quality of life. During the second period, the patients answered the same questionnaire when they were involved in their adult care. The psychological status of patients was assessed by the psychological general well-being (PGWBI). The PGWBI is a 22-item questionnaire which produces self-representations of intrapersonal affective or emotional states reflecting a sense of subjective well-being or distress, expressed by a summary score. The instrument measures components of psychological well-being such as anxiety, depressed mood, sense of positive well-being, self-control, general health and vitality. The closed-question questionnaire is a short list of items aimed at investigating the sense of security felt by patients against the pediatrician rather than the adult clinician.

## Results

See Table 1.

**Table 1:** 

PGWBI	anxiety sub scale	depressed mood subscale	positive well-being subscale	self-control sub scale	general health sub scale	vitality sub scale	overall PGWB score
**Before transition**	**13,09±6,61**	**10,91±3,42**	**8,45±3,56**	**9,64±3,26**	**9,00±3,10**	**11,91±3,94**	**64,50±17,52**

**After transition**	**12,45±5,22**	**10,64±2,54**	**7,82±3,19**	**10,64±3,47**	**10,55±3,21**	**11,00±4,40**	**63,45±17,04**

## Conclusion

Transition from paediatric to adult care for chronic diseases causes a little increase in anxiety but an improvement of self control and of general health. Patients report a fair amount of dissatisfaction, but the results of PGWB do not confirm their dissatisfaction. Even the results of the questionnaire on patient satisfaction show that patients feel secure with doctor, both before and after the transition.

## Disclosure of interest

None declared.

